# Supplementary material for: AI-determined similarity increases likability and trustworthiness of human voices
Source: PLoS One. 2025 Mar 5;20(3):e0318890. doi: 10.1371/journal.pone.0318890 (PMC11882051; doi:10.1371/journal.pone.0318890)
Supplement: Attachment — Submitted filename: reviewer_comments_trust_final.docx [file pone.0318890.s001.docx]

*Responses to Reviewer #1*

Kindly receive my comments on the on the manuscript entitled: “Do not trust your ears: Ai-determined similarity increases likability and trustworthiness of human voices” I think the title is attractive, but it needs, from my perspective, to be more scientific than popular. I suggest deleting the first part, “Do not trust your ears.”

That's true. The title has been changed.

The abstract lacks information on the approach and methods used.

Thank you for this comment. We have revised the abstract as follows:

*“*Modern artificial intelligence (AI) technology is capable of generating human sounding voices that could be used to deceive recipients in various contexts (e.g., deep fakes). Given the increasing accessibility of this technology and its potential societal implications, the present study conducted online experiments using original data to investigate the validity of AI-based voice similarity measures and their impact on trustworthiness and likability. Correlation analyses revealed that voiceprints – numerical representations of voices derived from a speaker verification system – can be used to approximate human (dis)similarity ratings. With regard to cognitive evaluations, we observed that voices similar to one’s own voice increased trustworthiness and likability, whereas average voices did not elicit such effects. These findings suggest a preference for self-similar voices and underscore the risks associated with the misuse of AI in generating persuasive artificial voices from brief voice samples.” (p. 2)

Line 60-63. … “Like fingerprints, the human voice can be used
61 to distinguish individuals from one another with a high degree of accuracy and gives insights
62 into the speaker's emotions and physical attributes”. The first part needs a reference.

We included the following references:

1.	Doddington GR. Speaker Recognition—Identifying People by Their Voices. Proceedings of the IEEE. 1985;73: 1651–1664. doi:10.1109/PROC.1985.13345

2.	Li H, Xu C, Rathore AS, Li Z, Zhang H, Song C, et al. VocalPrint: exploring a resilient and secure voice authentication via mmWave biometric interrogation. Proceedings of the 18th Conference on Embedded Networked Sensor Systems. Virtual Event Japan: ACM; 2020. pp. 312–325. doi:10.1145/3384419.3430779

Line 131. ..”Only one study has explored the relationship between voice similarity estimates by humans and an automatic speaker recognition system [37]. I think this is a very strong statement. You can extend your search to cover the following study:
Grágeda N., Busso C., Alvarado E., García R., Mahu R., Huenupan F., Yoma N.B. Speech emotion recognition in real static and dynamic human-robot interaction scenarios (2025) Computer Speech and Language, 89, art. no. 101666, Cited 0 times.
DOI: 10.1016/j.csl.2024.101666
Rágeda N., Busso C., Alvarado E., Mahu R., Yoma N.B. Distant speech emotion recognition in an indoor human-robot interaction scenario (2023), 2023-August, pp. 3657 - 3661
DOI: 10.21437/Interspeech.2023-1169

We appreciate the references. However, since they do not deal with the comparison of human and AI similarity assessment of voices, we have not cited them in the suggested place. However, as they investigate emotion perception, we have mentioned them in the introduction. Additionally, we added the following qualification:

“[…] to the best of our knowledge, there is only one study that has investigated the relationship between voice similarity estimates by humans and an automatic speaker recognition system [39].” (p. 5)

I suggest having the three research questions together as a list. This makes it clearer to the reader and more organized!

We included the following listing at the end of our introduction (p. 9):

- Based on the above considerations, we investigated
- whether the cosine similarity derived from the trained neural network correlates with human similarity judgments (Exp 1-3).
- whether speakers with prototypical voices are judged as more likeable and trustworthy (Exp 4).
- whether speakers with similar voiceprints to the corresponding participants are perceived as more likable and trustworthy (Exp 5).

Since “AI” is listed as a keyword and in the title, It is very necessary to have a paragraph about AI in general and then how it affects the voice and generates sounding voices and different platforms used.

Thank you for your advice. We have insert a brief paragraph at the beginning of the revised introduction.

“Artificial intelligence (AI) has become integral to modern life and is revolutionizing how people interact with technology and process information. From autonomous vehicles to personalized recommendation systems, AI's ability to analyze and replicate human-like behaviors profoundly impacts all industries. One particularly relevant application is in the field of speech technology, in which AI systems not only recognize and synthesize speech but also simulate individual voice characteristics. This capability opens new avenues for personalized interactions, such as matching voice assistants to a user's voice profile or augmenting that profile, illustrating the interplay between technology, identity, and human perception.” (p. 3)

It is very important to explain the perspective of how the authors understand/perceive the terms “likability and trustworthiness”. A comprehensive argumentation should be followed with proper references.

Thank you. We have restructured the introduction and introduced a section on likeability and trustworthiness:

“Likeability and trustworthiness are foundational attributes that significantly influence social interactions and relationships. Research has demonstrated that individuals judged more likable by others are more persuasive, often receiving preferential treatment and social support [27–30], and that similar others are also perceived as more likeable [31]. Similarly, trustworthiness is central in fostering long-term (business) relationships and ensuring effective collaboration, as it mitigates uncertainty, reduces the perceived risk in interactions and increases predictability [32–36]. From an evolutionary perspective, trustworthiness likely signals an individual’s reliability and cooperative intent, essential for fostering social cohesion and reciprocal behaviors within groups. Similarly, likeability facilitates social bonding by eliciting positive affect and reducing interpersonal tension, enhancing collaboration and mutual support. Thus, since these constructs are integral to social evaluation processes, the use of likability and trustworthiness as dependent variables is critical to understanding the impact of voice similarity.” (p.6)

The method part should be extended. The authors need to explain more about all the proceeds of experiments 1 and 2, 3, 4, and 5.

The sample also needs to be explained more (selection criteria, exceptions, exclusions, time of the experiment, is the gender balance considered?

As mentioned in the text, for each experiment, we recruited new German participants, with nationality being our only **selection criterion**.

Regarding the "time of the experiment," if this refers to the **duration**, the average length of each experiment is provided in the respective results section. If it refers to the **actual time of day**, the experiments typically occurred during daytime hours, although we did not have complete control over this aspect. Additionally, it is unclear how this information would enhance the readers' understanding, but we are open to providing further clarification if needed.

Regarding **gender balance**, we ensured equal representation in our first experiment. As no gender-related effects were observed, gender balance was not a concern in the subsequent experiments:

“Since previous research found evidence for an own-gender bias in the ability of voice identification [73] and gender differences in voice processing [74,75], we included participants gender as an additional factor. […] Numerically, female participants rated the similarity slightly higher; however, the inclusion of gender as an additional factor is not justified given the AIC values.” (p.13)

How did you approach the participants???

As noted, we used Prolific (i.e., an online platform in which participants complete experiments) to recruit participants, meaning we did not engage with them directly or in a personalized manner.

Did you inform them about the whole process? Did you provide a trial session for them before the actual experiments, or was it spontaneous??

Although we briefly mentioned the use of introductory trials, we have clarified and emphasized this more explicitly article. In the methods section of Experiment 3, we included the following additional details:

“After presenting three introductory trials, every 30th trial contained recordings of two different speakers, none of which came from the participant.” (p. 22)

In the methods section of Experiment 4, we included the following additional details:

“While the overall design of the experiment mirrored that of the second session in the third experiment —detecting sine tones to verify audio settings, performing three introductory trials, and judging 100 speakers — there were key differences.” (p.25)

I understand you have five experiments, each with a part for each method and result. However, this might be very confusing to the reader. I recommend you combine all methods in one part called “Materials and Methods”. Then, all results could be in one place under a title called “Results” but divided into parts as per experiments.

This is, of course, a valid suggestion and has already been considered by us. As the individual experiments build on each other and we believe that this makes the individual experiments easier to understand, we prefer the chosen structure. However, we have tried to emphasize differences between the methods.

Line 567: To me, this part is more discussion than a “general discussion.” If you want to keep it general, the logic says that you need to have another title called further/deep/focused discussion. Personally, I recommend having one title called “Discussion”.

Good point, we renamed it to discussion.

I recommend the following articles to be listed under the general text about “AI”:
10.3390/buildings14030786
10.3390/buildings14030781

We could not see how the papers mentioned are related to our manuscript. Even though we have added them to our list of articles to be considered in the future, we have decided to cite more broadly due to the lack of a current connection.

*Responses to Reviewer #2*

I would like to thank the authors for the innovative contribution to understanding the correlation between voiceprints and human perception. Using similarity assessments and d-vectors to generate speech that resembles a target speaker's voice, the authors concluded that cosine similarity is a valid measure for perceived similarity in this particular area with implications for cognitive research.

Many thanks for these words of encouragement.

In my opinion, the significance of the study is clearly stated and the manuscript is well-written although some sections need to be re-drafted. For instance, there is a lack of a "Background/Related Work" section which could better describe the state-of-the-art in the field under study. The Introduction section provides some hints but I would rather suggest to create a section that could describe the existing related work more comprehensively.

Thank you for pointing this out, we have restructured and partially rewritten the introduction and added, for example, information about similarity attraction effects (pp. 7-9).

The General Discussion could be slightly expanded with more implications for practice in terms of Text-to-Speech systems, while the Conclusion section is quite short and must be expanded accordingly.

Thank you very much for this suggestion. Throughout the manuscript we added information what the results might imply from a practical point of view. We also added a general limitations section and rewrote the conclusion section as follows:

“Our findings demonstrate that AI-derived cosine similarity measures effectively predict human voice similarity judgments and influence social evaluations. Across the first three experiments, we found significant relationships between the cosine similarity of voice embeddings and participants' similarity ratings, with a quadratic pattern emerging for judgments of other voices. This suggests that participants were particularly sensitive to highly similar and dissimilar voices, while intermediate similarity was more challenging to evaluate.

In Experiment 3, we extended this analysis to self-voice comparisons, revealing a general bias against perceiving other voices as similar to one's own voice. This bias likely stems from increased sensitivity to subtle differences in one's own voice or a Need for Uniqueness [88]. Comparisons to an internal mental representation or external audio recordings of the own voice yielded similar results, suggesting that the internal representation serves as a dominant reference.

Experiments 4 and 5 explored how voice similarity influences social perceptions such as likability and trustworthiness. Contrary to the beauty-in-averageness effect found in visual stimuli, we found no evidence that average voices were perceived as more likable and only weak effects on trustworthiness. However, voices similar to one's own were judged as both more likable and trustworthy, supporting the similarity-attraction hypothesis and the influence of implicit egotism.

Our results highlight the potential of AI-generated cosine similarity as a tool for understanding voice perception. While individual effects were modest, the consistency of the findings underscores their practical relevance for voice-based technologies like personalized voice assistants or synthetic speech systems. Future research should focus on refining models for highly similar voices, exploring cross-linguistic generalizability, and addressing the ethical implications of voice similarity manipulations. This study advances our understanding of voice similarity's role in cognition and social interaction by bridging human perception and AI-driven voice representations.” (pp. 33-34)

Regarding the methodological issues, the recruitment occurred between March 2021 (Experiment 1) and November 2021 (Experiment 5), which means that the data presented here has more than 2.5 years already when considering the last experiment conducted.

This is of course an appropriate observation. However, there seems to be no reason why we would expect our experiments not to replicate if we would rerun them today.

The authors should also provide more details about the recruitment process on Prolific (e.g., total amount of USD paid per HIT). A better description of the interface and tasks presented to the participants would be helpful.

We think this is a very good suggestion. We included information of the received payment in every participants section:

Experiment 1: “Participants received £3.45 for their participation in the study.”

Experiment 2: “Participants received £4.36 for their participation in the study.”

Experiment 3: “Participants received £5.00 for their participation in the study.”

Experiment 4: “Participants received £2.82 for their participation in the study.”

Experiment 5: “Participants received £3.76 for their participation in the study.”

Additionally, we revised the description of the experiments as follows:

“Since our experiments were conducted online on pavlovia.org (https://pavlovia.org/) using PsychoPy [69], we checked whether participants had a working audio setup at the beginning of the experiment: We presented a short text in which we informed them to count sinus tones. After presenting four sinus tones with an inter-stimulus interval of 1s, participants should indicate on a slider (ticks on 0,1, 2,3,4 and 5) how many sinus tones they were hearing. If they failed the test, the experiment was concluded. If they passed the detection task, three introductory trials were presented that had the same structure as the regular trials; audio recordings from two different male speakers were presented sequentially, with an inter-stimulus interval of 1s. While audio was played a headphone icon was depicted. After hearing both voices once, the participants were asked to rate the dissimilarity by adjusting the slider on an unmarked continuous rating scale (range: little dissimilarity - great dissimilarity).” (p. 12)

Minor issues: The first part of the title needs a question mark (?)

We deleted the first part of the title.

I usually have doubts when authors claim that there is only one study addressing a certain aspect (e.g., "only one study has explored the relationship between voice similarity estimates by humans and an automatic speaker recognition system [37]").

Thank you for your comment. We claimed to bold, so we changed it as follows:

“Beside research on performance differences between human and speaker recognition systems [23], to the best of our knowledge, there is only one study that has investigated the relationship between voice similarity estimates by humans and an automatic speaker recognition system [24]” (p. 5)

Fig. 2 is labelled as showing an "Illustration of the results of Experiment 3" but it provides insights for the Experiment 2

Thank you, we changed it to “Experiment 2”.

Instead of naming sections as just "Experiment 1…2…3…", I would kindly suggest to create section titles with the main purpose of each experiment.

Thank you for this helpful suggestion. We changed it as follows:

Experiment 1: The relation between AI and human similarity judgments

Experiment 2: The reliability of human similarity judgments

Experiment 3: Similarity judgments in relation to the own voice

Experiment 4: The beauty in average voices

Experiment 5: The attraction towards similar voices

Acronyms like LSTM, AIC, and AI should be described in the first time they are mentioned in the text.

Done.

via prolific -> via Prolific (insert a link to the crowdsourcing platform as footnote)

Since PLOS ONE does not accept footnotes, we added the following intext hyperlink: ([https://www.prolific.com](https://www.prolific.com/)).

MOSNet and MOSNET -> MOSNet (uniformity)

Done.

"[…] aggregated dataset compared to the first Experiment" -> "[…] aggregated dataset compared to the first experiment"

Done.

*Responses to Reviewer #3*

The present manuscript aims to explore the utility of voice similarity as measured by a neural network as a proxy for human perception of voice similarity as well as how this similarity might then guide perceptions of person likeability and trustworthiness. Across 5 experiments, the manuscript presents some evidence that neural network similarity measures could approximate human similarity measures to an extent when comparing unfamiliar voices (Exp. 1-2) or one’s own voice to unfamiliar voices (Exp. 3). There is also some indication that voices considered more typical (as measured by a neural network) are perceived somewhat more trustworthy (Exp.4) and that voices measured to be more similar to one’s own voice are also perceived as more likeable and trustworthy (Exp. 5). This work undoubtedly asks some interesting and very timely questions but I do have some concerns about the strength and generalisability of the reported effects. Please find my major and more minor comments detailed below.

A large part of the Introduction is overly technical which could be particularly challenging for a broader audience. Personally, I am a behavioural scientist (not a computer scientist or a linguist) and I could not make sense of all the terminology used throughout. Also, more clarity is needed when reporting previous findings – most of the literature presented here was based on computer-based estimations, not behavioural ones but this is not made explicitly clear in the text. For example, at the start, it is stated that human voices can be used to distinguish individuals from one another – this is actually a very challenging and error-prone task for human observers (who have even been shown to struggle recognising their own voice). Same is true for the examples provided for recognising stress, emotions, demographics, etc. – they are mostly based on computer measurements. While human observers can also detect these in other’s voices, it is worth highlighting the perspective the authors are taking here to avoid any confusion.

We have adapted the introduction emphasizing on behavioral findings. We are also behavioral scientists and have probably overdone it with the technical terms. Nevertheless, we can't avoid describing the technical terms, however, we tried to link that to behavioral descriptions when possible.

The overview presented at the end of the Introduction lacks clarity and does not help to get a good idea of the overall structure of this work. For example, it is not stated what the differences between Experiments 1-3 are. It might be clearer to simply describe each of the 5 experiments briefly.

Thank you for pointing this out. We hope that the restructuring and supplementation of our introduction will provide more clarity. In particular, we now provide a list of the research questions as well as their links to the individual experiments:

“Based on the above considerations, we investigated

- whether the cosine similarity derived from the trained neural network correlates with human similarity judgments (Exp 1-3).
- whether speakers with prototypical voices are judged as more likeable and trustworthy (Exp 4).
- whether speakers with similar voiceprints to the corresponding participants are perceived as more likable and trustworthy (Exp 5).” (p. 9)

The authors provide no clear hypotheses and there is no justification as to why they are expecting quadratic relationships. Was this based on some previous research where such quadratic relationships were reported?

As stated in our preregistration, we did not collect any data beforehand. We included a quadratic model because human judgments, particularly those based on perceptual features like voice similarity, often show non-linear trends. We added the following statement:

“We included a quadratic model because human judgments, particularly those based on perceptual features like voice similarity, often show non-linear trends [73]. This approach accounts for potential non-linear relationships between the cosine similarity of voice embeddings and human similarity judgments. For instance, individuals may perceive two voices as more similar up to a certain point, but after that, additional increases in cosine similarity might not yield proportional increases in perceived similarity. This suggests diminishing or varying returns on perceived similarity as cosine similarity increases.“ (p. 13)

There are some methodological clarifications needed throughout:

What online platform was used to present these studies?

We added the following information:

“Since our experiments were conducted online on pavlovia.org (<https://pavlovia.org/>) using PsychoPy [49]“ (p. 12)

Is there any particular reason why only male voices were used in Exp. 1?

This is primarily a result of practical considerations and the fact that it was our first-ever experiment with such data. However, we also wanted to obtain the finest possible gradation of cosine values. We have supplemented the following paragraph accordingly:

“Since our dataset included more male than female speakers (approximate ratio of 3:1), and because this was our first experiment using this type of data, we aimed to achieve a wide range of cosine similarity values with high granularity. We used only male speakers in this study to simplify the experimental design and ensure consistent conditions.” (p. 11)

From my reading of the text, it seems like each pair of voices was rated by 2 participants only – this might not therefore lead to a very reliable, stable and generalisable behavioural similarity index.

Thank you for your comment. You are correct that each pair of voices was rated by two participants, one male and one female. This design intentionally controlled for potential gender-related differences in similarity judgments and ensured these differences did not confound our results. This initial experiment aimed not to establish a stable or generalizable behavioral similarity index for specific voices but to validate that our methodology reliably captures perceptual differences and excludes biases arising from individual or specific voices. By employing a random sampling approach, we aimed to lay the groundwork for future experiments that build on this controlled and balanced design.

Were participants able to replay the pairs of voices? - How was the unmarked continuous rating scale used to provide a numerical similarity measure?

Participants were not able the replay the voices. Additionally, a rating scale is a stimulus component in PsychoPy that logs the responses in numerical format with 9 decimal points. We added the following information:

“After hearing both voices once, the participants were asked to rate the dissimilarity by adjusting the slider on an unmarked continuous rating scale (range: little dissimilarity - great dissimilarity).” (p. 13)

A power analysis is provided for Exp.2 but none of the others. It is also worth specifying the exact effect size used for this calculation. Is this the authors’ estimate of the relationship between first and second ratings or the relationship between AI-measured and behavioural voice similarity?

This question is of course completely justified. We have added the following information to the methods sections of experiment 1, 2, and 3:

“G*Power [78] was used to calculate the necessary sample size for the Correlation: Bivariate normal model test as an approximation for the non-parametric Spearman rank correlation test that was used to calculate the test-retest reliability.” (p. 16)

“We chose a sample size of 100 participants for our first experiment as a practical starting point. This sample size provided a balance between feasibility and statistical power, allowing us to evaluate the relationship between cosine similarity and human similarity judgments while accounting for individual variability.” (p.11)

“To ensure statistical consistency across experiments and facilitate meaningful comparisons, we used the same sample size of 100 participants in Experiment 3 (and the remaining experiments) as in Experiment 1. This approach minimizes potential discrepancies arising from differences in statistical power.” (p. 21)

In Exp.2, participants heard the voice pairs a second time but in the same order they first heard them in. The authors argue that this is to avoid variance from individual randomization but I’m concerned that this approach might artificially increase the correlation between first and second ratings, especially if participants can recall the pattern of responses from the first rating.

Sorry for the misunderstanding, but we already had this information in the methods part of experiment 2:

“The order of the speaker pairs was altered in the second part of the experiment but was the same for all participants.” (pp. 16-17)

In Exp.3 – were participants asked to indicate to what extent the recordings of their own voice sounded similar to their representation of their own voice?

No. They were just instructed to judge the similarity. We added the following information:

“In both groups, we simply asked the subjects to rate the similarity of the other person's voice to their own voice, and therefore did not mention that their own voice might have an internal representation.” (p. 22)

As stimuli were gender-matched in Exp.3, does that mean that participants who identified as diverse or did not specify their sex, were excluded?

No, gender matching was accomplished using the information provided by participants on Prolific. We also collected demographic data through Qualtrics, which primarily served to obtain participant consent and redirect them to their individually generated experiments on Pavlovia. We chose not to exclude participants based on gender to avoid discrimination. However, there are so few of them, so the effect would not change if they were excluded. Nevertheless, this information is included in our data which we made available on figshare (<https://doi.org/10.6084/m9.figshare.21022081.v2>), allowing you to consider it. Additionally, we have added the following details to the methods section of Experiment 1:

“Basic demographic information was collected via Qualtrics (<https://qualtrics.com>) in all experiments.” (p. 11)

Were participants explicitly instructed that they will be hearing a recording of their own voice on each trial?

No, they were simply asked to “rate the dissimilarity of the presented voice in comparison to their own voice. We added the following information:

“If they were assigned to the external representation group, on each trial, participants were randomly presented with one of their five audio recordings, followed by another person's audio sample – without them having been instructed to listen to their recordings beforehand.” (p. 21)

How many trials did participants complete in Exp.3?

We added the following information:

“In the second session, after checking the audio setup, in 100 trials the participants were asked to rate the dissimilarity of the presented voice in comparison to their own voice.” (p. 21)

Some further clarification is needed about the attention check used – it is stated that participants were asked to detect trials where voices were from two different speakers. Was that not true in all trials? My understanding is that voices from two speakers were presented at every trial.

The design of the attention checks differed across the experiments. We described this in each section. Regarding Experiment 3, you are right that the description lacks clarity. We revised the sentence as follows:

“After presenting three introductory trials, every 30th trial contained recordings of two different speakers, none of which came from the participant.” (p. 22)

How many speakers were used in Exp.4?

Thank you for highlighting the missing information. We incorporated the following information:

Experiment 3:

“Ten speakers were randomly selected from each category, resulting in a total of 100 speakers.”

Experiment 4:

“To obtain the highest possible variance in typicality, we assigned each mean cosine similarity value to one of ten similarity categories and randomly selected ten speakers from each category, resulting in a total of 100 speakers.”

Experiment 5:

“As in the third experiment, the first session was used to record voice samples, compute the voiceprints, and sample 100 same-gender speakers from our dataset with a wide variety of cosine similarities.”

The authors argue that average faces are perceived as more attractive but it might be worth pointing out that this is not always the case. For example, Perrett et al. (1994, Nature) present some evidence against the averageness hypothesis.

Perrett et al. (1994, Nature) do not entirely refute the beauty-in-averageness effect but emphasize that average faces may not represent the peak of attractiveness. However, your observation is highly valuable, and we have incorporated the following clarification:

“Our investigation was motivated by the concept of the beauty-in-averageness effect [42,43], which suggests that average features are often perceived as more attractive – even though they may not be optimally attractive [62].” (p. 24)

Looking at the data made available by the authors, it seems like some pairs of voices have a negative cosine similarity – this might be worth mentioning in the text together with how to interpret these negative values.

Since cosine similarity values range from -1 to 1, negative values are not inherently unusual; they simply indicate lower similarity between the voices. To clarify this, we provided the following explanation:

“The similarity of two voices can be compared by calculating the cosine similarity of two feature vectors, yielding values ranging from -1 to 1.” (p. 10)

How was the median correlation calculated?

We hope, that the addition of the following information may answer your helpful question:

“The median of the individual Spearman Correlations between the encoder’s cosine similaritiy values and participants similarity ratings was Mdn r_s_ = 0.37 (Q_1_ = 0.28, Q_3_ = 0.43), indicating a moderate relationship.” (p. 13)

The main effect reported in the paper – the relationship between human- and AI-based measures of voice similarity seems rather inconsistent in size. In fact, the median correlations reported decrease with every next experiment.

Indeed, there is a decrease in correlations from experiment 1, 2 and 3. While we have discussed this in the submitted version of the manuscript, we revised the discussion of this matter throughout the article. See examples below:

“The relationship, however, was slightly less pronounced than in Experiment 1. This may be explained by the limited stimulus material required to measure the reliability scores as well as the reduced number of trials to keep the experiment within reasonable boundaries.” (p. 17)

“As we noticed an overall decrease in similarity ratings when participants compared voices to their own voice, we conducted post-hoc Tukey-Kramer tests to investigate differences across the three experiments. Significant differences emerged between the average slider responses: *M*_2_ - *M*_1_ = 0.03, *t =* 5.49, *p* < .001; *M*_3_ - *M*_1_ = -0.08, *t =* -18.80, *p* < .001; *M*_3_ – *M*_2_ = -0.11, *t =* -20.44, *p* < *.*001. These findings suggest a consistent bias where participants are less likely to judge voices as similar to their own. […] The lower similarity ratings may stem from a *Need for Uniqueness* [89], where participants hesitate to identify other voices as similar to their own. Alternatively, heightened familiarity with one's own voice may increase sensitivity to subtle differences, leading to underestimation of similarity.” (p. 23)

I also worry about the seemingly low within-rater consistency – it might indicate that people are not able to do this reliably enough. What is more, nothing is said about the linguistic content of the recordings. I’m assuming that different speakers had different utterances – therefore it might be that perceivers remember the utterance which can then artificially increase their consistency.

Thank you for pointing that out. Of course, there is nothing we can do about the low within-rater consistency, which most likely shows the difficulty of this task. However, this also speaks against the idea that participants only remembered the linguistic content; otherwise, the within-rater consistency would have to be higher. In addition, they would have to remember the content and the provided rating. Although this could be possible, it seems unrealistic to us.

Regarding the linguistic content, we added the following information:

“To train the network, we used the German subset of the Common-Voice dataset (<https://github.com/mozilla/common-voice>), Distant-Speech (<https://www.inf.uni-hamburg.de/en/inst/ab/lt/resources/data/acoustic-models.html>), LibriVoxDeEn [68], and the German subset of the VoxForge dataset (<http://www.voxforge.org/home>). These datasets contain linguistically diverse material. The linguistic content ranges from simple phrases to complex sentences, covering a broad spectrum of phonetic, lexical, and syntactic structures in German. The combined datasets consist of approximately 1,000 hours of spoken audiobooks, and Wikipedia articles read aloud by about 10,000 non-professional speakers. Audio files not already cut at sentence boundaries were cut at the appropriate points.” (p. 10)

I do not think it is appropriate to apply an attenuation correction due to the low perceiver reliability – low reliability means that perceivers might not be able to do this task with high levels of consistency, not that the correspondence between human and AI similarity measures might be overestimated.

Thank you for pointing this out. Although we are convinced that not applying such a correction would lead to an underestimation of the correlation and would prefer to retain it, we have added the remark that the calculated value should be regarded as the upper bound of the correlation:

“The observed reliability is crucial in understanding the correlation between AI-generated cosine values and human similarity judgments. Any error or inconsistency in human judgments (as suggested by the reliability value less than 1) can attenuate or reduce the observed correlation. This means that the true correlation would likely be higher in the absence of such errors. Therefore, the obtained correlation between AI ratings and human judgments underestimates the actual strength of this relationship due to the influence of measurement error inherent in human judgments. Considering this reliability, our attenuation correction suggests that the true correlation is stronger than what is directly observed – even though the correlation values calculated by the attenuation correction are to be regarded as upper bounds.” (pp. 18-19)

I realise that this might be a personal preference, but I would not advise describing an effect with a p value of .078 as marginally significant.

We are also bothered by this expression and have debated whether we should even call it that. We are relieved that you feel the same way. We have changed it as follows:

“An ANOVA contrasting the quadratic with the linear model just missed the threshold of significance, *F*(1,7) = 4.27, *p* = .078.” (p. 22)

In the Discussion, authors argue that a potential problem with voice averages (composites) is that they might be representative across age. Is their suggested alternative stable across time?

We may not understand your comment correctly. We are simply pointing out in the discussion that the harmonics-to-noise ratio decreases with age, and that voice composites generally produce a higher harmonics-to-noise ratio, which is generally associated with higher vocal attractiveness.

*Responses to Reviewer #4*

The authors present a series of experiments in which they test whether an AI AI-based voice similarity measure (voiceprint) corresponds to human similarity measures and whether voice similarity influences the voice’s trustworthiness and likeability. They report a correspondence between the voiceprint and human ratings as well as (in one experiment) an increase in perceived trustworthiness and likeability with similarity to one’s own voice. The topic is interesting and timely, and the experiments are well conducted and analyzed.

Thank you very much.

While I applaud the authors for this, I see some serious flaws in the paper which can partly easily be mended, but partly would need at least a very thorough rethinking of the argument. It is not impossible that they are due to the fact that I am not an expert in the topic. But even then, I think they show that the authors are not making themselves sufficiently clear to non-experts. In fact, I accepted the review because I assumed trust would be a central theme; here I would have been an expert. The first part of the title misled me (and the abstract did not correct this). So, I would suggest a different title – and one that is less loud, by the way.

We sincerely apologize if we have come across as a little pretentious. We have changed the title.

The authors claim relevance because of the introduction of a new methodological tool and the possibly important results and speak of a broad appeal of their results. This is not implausible, but this part specifically is not well developed. I would expect more precise arguments (or less strong claims).

Thank you for pointing this out. However, we are not interested in the tool itself, but in its’ potential to generate customized voices. This technical potential is new and the observed effects can also have a significant social impact due to their prevalence. For instance, a purchase recommendation by a similar voice leads to a 2% higher purchase rate is a large absolute difference with a corresponding user base. To point this out, we added the following information at the end of our discussion:

“Even though the effect may have been relatively small in our experiments, due to the widespread use of voice assistants and the more elaborate methods available to large technology companies, the impact can be tremendous in absolute terms. This applies not only to the use of user-adapted voices to make interactions with the assistants more attractive but also to the impact on advertising messages and political propaganda.” (p. 32)

The introduction is disorganized; the reader must construct the line of reasoning himself, understanding a series of scattered defined technical terms and finally figuring out what is actually the core of the later examination, the technical terms or human perception. Statements on the social relevance of the topic are thrown in, but not very integrated (and rather strongly formulated). It seems to me that the Introduction would have to be totally rewritten, and I am not sure that a tightly knit Introduction would lead to exactly the present experiments as an appropriate answer to the questions. I especially wonder whether the two parts – the test of the voiceprint and the test of the influence of similarity – need to be published together. Neither part is very strong, but putting them together in one paper does not make them stronger. Also, the research questions should be better developed and better derived from the introductory paragraphs.

You are absolutely right that the introduction needed an overhaul. We hope that our revision has taken into account the points of criticism you raised.

I would make effect sizes and explained variance a much more prominent part of the presentation of results and the interpretation and conclusion. The relatively strong claims about the practical relevance of the study that are interspersed throughout the text only make sense if combined with a clear estimate and discussion of the practical relevance.

We have adapted the results sections and hope that this meets your expectations. For example, we rewrote the following paragraph for Experiment 5:

“We compared an intercept-only model, a linear model, and a quadratic model for both the likeability rating as well as the trustworthiness rating. Weighting using the AIC values in Table 6 showed a quadratic relationship between the voice similarity and likeability ratings (intercept: 0.47, 95% CI [0.45, 0.49], t(111) = 46.70, p < .001; cosine: 0.11, 95% CI [0.05, 0.18], t(8863) = 3.55, p < .001; mean cosine2: 0.17, 95% CI [0.04, 0.30], t(8864) = 2.59, p = .009; Rc2 = 0.19, indicating that 19% of the variance in likeability ratings was explained by the model). The median of the individual Spearman Correlation between cosine similarities and likeability ratings was Mdn rs = 0.15 (Q1 = 0.05, Q3 = 0.25), which, while modest, demonstrates a consistent positive relationship.” (p. 13)

What is a “fair amount” of explained variance in this context?

We replaced this interpretation with the exact percentage value.

I wondered whether some of the more technical parts could be moved to a subsection in the Introduction. As such, it is one major source that distracts from a psychological argument in the Introduction.

We have revised the introduction and hope this will make it easier for the reader to understand the article.

For all experiments, there are long, dense, and often unexplained sections describing results and comparatively short sections discussing them. Methods should be better justified (sometimes explained), and the explanation what the results mean and how they are limited should be substantively expanded.

Thank you very much for pointing this out! After rereading our manuscript with this criticism in mind, we agree. We modified the text and added multiple explanations. For example:

“Taken together, these results demonstrate that the quadratic relationship between voice similarity and ratings of likeability and trustworthiness accounts for a substantial proportion of the variance. These findings suggest that while individual effect sizes are modest, the overall fit of the models underscores the practical relevance of voice similarity in shaping social perceptions. Indeed, these findings support the similarity-attraction hypothesis [44,45], in such that voices similar to one’s own are perceived as more likable and trustworthy. The quadratic relationship suggests that the effect is stronger for higher levels of voice similarity. This effect likely stems from implicit egotism [47–49] suggesting that individuals evaluate self-associated traits positively, or from social identity processes, in which perceived similarity fosters a sense of connection or group affiliation [55]. These results highlight the potential for AI systems to exploit similarity effects in personalized technologies, such as voice assistants, to influence user perceptions and behavior.” (p. 28)

We also added a General limitations chapter:

**“**While our study provides valuable insights into the relationship between voice similarity and cognitive evaluations, several limitations must be acknowledged.

A major limitation of this study arises from the wide range of voice similarity values that we were able to investigate. By including pairs of voices from a wide range of the similarity spectrum, we were able to ensure a solid understanding of the overall pattern; however, this broad spectrum may have diluted specific effects that are particularly pronounced in highly similar voices. Future studies should focus more on speakers with high cosine similarity values to better capture the nuances and practical implications of judgments in this critical range. This limitation also reflects a trade-off between experimental control and ecological validity. While our design provided valuable insights into general trends, focusing exclusively on highly similar voices may provide more precise and application-oriented results.

The use of open-source datasets, while providing a wide range of speaker voices, also introduced variability in audio quality, articulation, and linguistic content. These factors may not only have influenced participants’ judgments but reduced the internal validity of the experiments. Future work should employ more controlled datasets or systematic manipulations of stimulus properties to reduce potential biases.

Our study emphasized static voice similarity judgments based on pre-recorded audio. Dynamic aspects of speech, such as conversational context, prosody, or situational factors, were not considered. These elements are likely to influence perceptions and warrant exploration in future research.

Finally, the online nature of the experiments presents challenges such as variable listening environments and participant compliance. Although attention checks and control trials were implemented, these measures cannot fully account for potential distractions or technical issues encountered by participants during the study.” (pp. 32-33)

In several places it seems as if the authors – post hoc – explain away unexpected results (changes between experiments, low correlations) (e.g. 11 and 13). However, the respective objections are highly relevant for their study and its interpretation.

Our aim is not to reinterpret or overstate the results, but rather to engage in their interpretation as part of our task—an effort that is inherently post-hoc. We acknowledge that there are valid critiques of our experiments, including the limitations of the material used. Unfortunately, we were unable to collect or produce the extensive amount of speech samples needed to train the encoder while maintaining sufficient control over the material. Therefore, we relied on the resources available to us. Nonetheless, it remains necessary for us to address and interpret aspects such as the decreasing correlation values, which requires careful examination of the results.

Additionally, it is important to note that our research operates within a relatively new and exploratory field, where outcomes can be more challenging to predict. We want to emphasize that our intention is not to overstate our findings. Transparency, following the principles of open science, remains a top priority within our institutes.

However, we reprhased the results parts of experiment 1 (originally on page 11):

“While the association between similarity values and similarity judgments appears modest in the analysis with mixed models, the analysis with aggregated data suggests that the relationship warrants attention. This finding is noteworthy given the potential limitations of using stimuli derived from open-source datasets. Factors such as varying audio quality, speakers' articulation proficiency, and the semantic content of audio clips could have influenced evaluations. At the same time, the diversity of the stimuli may have contributed to the ecological validity of incidental similarity evaluations.

It is also important to consider that assessing the similarity of two voices based on just 5 seconds of random samples is inherently challenging. The quadratic relationship observed in the data indicates that these challenges were particularly pronounced when participants evaluated speaker pairs with moderate to low cosine similarity values. These complexities, and their potential implications for interpreting the results, will be explored further in the General discussion.” (p. 15)

Similarly, for experiement 2 (originally on page 13):

“This may be explained by the limited stimulus material required to measurethe reliability scores as well as the reduced number of trials to keep the experiment within reasonable boundaries.” (p. 17)

The General Discussion contains at least one argument that should have been considered pre-hoc instead of post-hoc (p. 23, threshold an ecological validity).

We may disagree with this comment. Firstly, this observation is an assumption based on our experimental results. Secondly, this finding would not change the design of our experiment, because otherwise we would have to set this threshold relatively arbitrarily. And thirdly, this consideration does not change the conclusion that matching voices are evaluated more positively. On the contrary, the inclusion of very dissimilar voices should reduce bias.

So, considering PLOS ONE’s criteria for Reviewing papers, there are (partly substantial) weaknesses in
• stating the main claims of the paper and showing how significant are they for the discipline,
• in supporting the claims with the data, or, more precisely, in discussing the amount of support in a balanced way
• in accessibility for non-specialists and organization of the paper

As the study shows much potential, a thoroughly revised version (maybe including new experiments) could be appropriate for PLOSE ONE.

While we are not able to conduct further experiments, we sincerely hope that the revised version may find your approval.

The Introduction contains a sentence that includes “Forecasts predict that by 2024 …”. As we are already well into 2024, it would be appropriate to compare the forecast to reality here.

This reference has been removed from the revised version of the introduction.

p. 7: It is unclear to me, how a research question can extend findings.

We removed this formulation.

p. 9: Here, a slider is introduced suddenly. Could be explained better in the methods section.

You are right, the term “slider” is now also used in the methods section:

“After hearing both voices once, the participants were asked to rate the dissimilarity by adjusting the slider on an unmarked continuous rating scale (range: little dissimilarity - great dissimilarity).” (p. 12)

Experiment sections should not end with new (secondary) information (p. 15).

Thank you for this feedback. We have revised this section as follows:

“The findings confirm that AI-derived cosine values are predictive of human similarity judgments, though their strength varies depending on dataset restrictions and individual variability. Stronger correlations at extreme similarity values underscore participants' difficulties in making reliable judgments for speaker pairs of average similarity. This pattern complements the quadratic relationship observed in Experiment 1, where the dissimilarity of speaker pairs posed challenges for nuanced ratings. Overall, the results reinforce that similarity judgments are inherently challenging and prone to individual differences, particularly in moderate similarity categories.” (p. 20)
